# The Outcome of Cardiac Hydatid Surgery in The Iraqi Center of Heart Diseases

**DOI:** 10.12688/f1000research.172453.1

**Published:** 2026-01-16

**Authors:** Maath Mohammed Muhsin, Noor Hussain Abady, Aminah Hilal Khallaf

**Affiliations:** 1 Pathology, University of Fallujah, College of medicine, Fallujah, Iraq; 2 Surgery, University of Fallujah, College of medicine, Fallujah, Iraq; 3 Gynecology, Nu'man teaching hospital, Baghdad, Iraq

**Keywords:** Cardiac hydatid cyst, Echinococcosis, Cardiac surgery.

## Abstract

**Background:**

Cardiac hydatid cysts are uncommon presentation of heart disease that most often affects the left ventricle of the heart. The clinical picture may be quite different and may include arrhythmias and myocarditis up to a potentially life-threatening embolism of the cystic contents in cases of rupture into a cardiac chamber. Early and proper diagnosis is very important to avoid serious complications, such as infection and rupture of cysts. Even though medical treatment can bring certain control, surgery is the preferred type of treatment, which has good results and a low possibility of recurrence in cases where it is performed early.

**Methodology:**

This retrospective case-series study examined five patients diagnosed with cardiac hydatid cysts between January 2018 and July 2024. All cases were handled by a qualified cardiac surgeon at Al-Anbar Governorate in Iraq. The effectiveness and safety of surgical management were evaluated based on clinical information, radiographic findings, surgical procedures, and post-operative experiences.

**Results:**

All five patients successfully underwent successful excision of the cardiac hydatid cysts. In all cases, the post-operative course was uneventful, except for one patient who developed significant post-operatve bleeding requiring re-exploration. There were no deaths, and high-quality clinical recovery was achieved. No incidence of cyst rupture, secondary infection, or cyst reccurrence was reported during the follow-up period.

**Conclusions:**

Although uncommon, cardiac hydatid cysts should be included in the diagnosis of cardiac masses, particularly in endemic areas. Echocardiography and high-tech imaging should be used to diagnose heart disease early to prevent severe complications. Surgery has been the pillar of treatment as it offers high success rates with low risk in case it is performed in a timely and careful manner. The positive results in this series prove the necessity of timely surgical referral and multidisciplinary treatment.

## Introduction

Hydatid cysts are parasitic infections mostly caused by *Echinococcus granulosus* and are mostly observed in the liver and lungs. *Echinococcosis* is a human infection caused by the larval stage of any species of the *Echinococcus granulosus* complex, E. multilocularis, or *E. vogeli.* *E. granulosus* complex parasites that cause unilocular cystic lesions are common in areas where livestock are kept in close proximity to dogs. ^1^

*Echinococcus granulosus* has a 2-host life cycle. Dogs and other canines are definitive hosts, whereas sheep and other herbivorous animals are intermediate hosts. Humans serve as an incidental intermediate host (dead end), acquiring infection by ingestion of food contaminated with feces from dogs harboring *E. granulosus* eggs. ^2^

Eggs develop into larva (hydatid cyst) In duodenum, It pierces through the intestinal wall and gets into the portal circulation and is transported to the liver or lungs or seldom to other organs. The host immune response attempts to eliminate the parasites. Thus, an inflammatory reaction occurs around the areas where the parasite is lodged; the host immune reaction may kill many of the parasites, yet some of them may avoid being killed and grow to become hydatid cysts, surrounded by fibrous connective tissue, and become fluid-filled bladder-like cysts called hydatid cysts, which are most frequently located in the liver (60-70%, in the right lobe) or lung (20-30%), but can occur in any organ such as the spleen and kidney (35%), brain and heart (11.5%), and also in bones in rare cases. ^2^

Through the coronary arteries, the hydatid cysts may find their way to the heart, usually getting trapped between the myocardial layers. Secondary cardiac infection can occur after a primary hydatid cyst ruptures into the pericardial cavity. Such secondary cysts are initially superficial and subepicardial but can later infiltrate the myocardium. It is worth noting that hydatid cysts of the pericardium are always caused by rupture of a primary cyst, which is practically located almost entirely in the cardiac tissue. ^3^

The hydatid cyst reaches the heart via the coronary arteries and is located inside the myocardial layers. Another cardiac focus can occur following rupture of a primitive hydatid cyst in the pericardium. Secondary cysts are then superficial and subepicardial, although they may extend to the myocardium hydatid cysts in the pericardium; however, they are consistently subordinate to the rupture of a primitive hydatid cyst, which is typically located in the heart region. ^3^

Patients who have an abnormal heart shadow on a **chest x-rays (CXR)** and have come out of an endemic region should be suspected of the disease and diagnosed. The cyst tends to increase in size and, therefore, compresses the surrounding myocardium. It causes displacement of the coronary vessels, rhythm disorders, and mechanical interference with atrioventricular (AV) valves and ventricular function. Echocardiography is the preferred imaging technique for the diagnosis of cardiac hydatidosis.

CT can be performed to ensure diagnosis as well as to rule out liver, lung, and brain hydatid in cases of doubt. The treatment of cardiac echinococcosis is based on the size, location, and symptoms of the cysts and the general condition of the patient. Conventional definitive treatment includes surgery. As an adjunctive therapy, albendazole, which is active against Echinococcus, should be used several days before resection and several weeks later. ^4^

## Treatment

Prior to the administration of anti-helminthic drugs, surgery was the only option to treat Echinococcosis. Although it is still the most widely used treatment method, surgical intervention is associated with significant risks, among which there is a significant mortality rate of as high as 2 percent in some series and repeat operations with augmented risk, with a morbidity and recurrence rate of 2 per cent to 25 per cent.

Anti-helmintic agents, albendazole, and mebendazole have demonstrated efficacy against cystic echinococcosis. Albendazole is the drug of choice because it has better systemic absorption and penetration into hydatid cysts than mebendazole.

Medical treatment alone is indicated for **inoperable cysts** because of the location or medical condition of the patient, and in **multiple organ cysts**, medical treatment is used preoperatively and postoperatively to prevent and reduce the risk of recurrence. ^5^

This study was concerned with five cases by one surgeon experience, all of whom lived in the same governate, in rural (one of five) and urban areas, and had no contact with animals (sheep), each of which had a different presentation. All patients underwent cardiac surgery, and the follow-up program was improved.

## Aim of study

Aim of this study was to recognize different presentations of cardiac hydatid cysts, especially in endemic areas with high suspicion, as early diagnosis and treatment lead to the avoidance of complications of cardiac hydatid cysts (infection and rupture).

## Patients and methods

**Figure 1.**
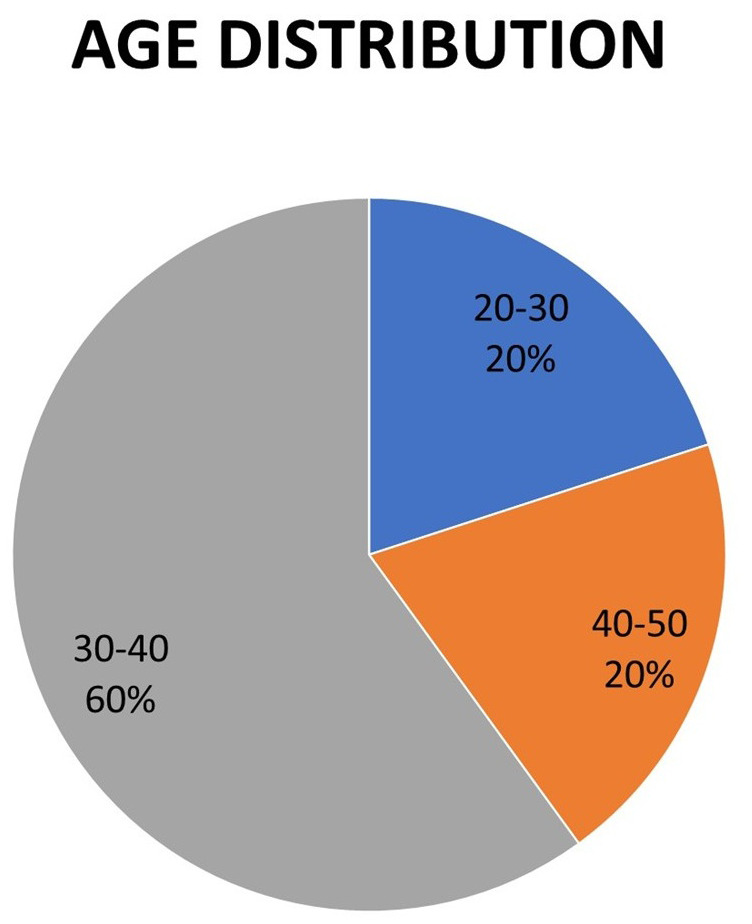
Age distribution.

This was a retrospective case series study of five cases of cardiac hydatid cysts from January 2018 to July 2024, one in 2018, another one in 2020, two in 2023, and the last in June 2024. The age distribution was 20-50 years as shown in Figure 1 and 4:1 female: male ratio. All patients were referred by a cardiologist to a cardiac surgeon, and all patients underwent open heart surgery through median sternotomy, cardiopulmonary bypass, and cardiac arrest.

**Figure 2.**
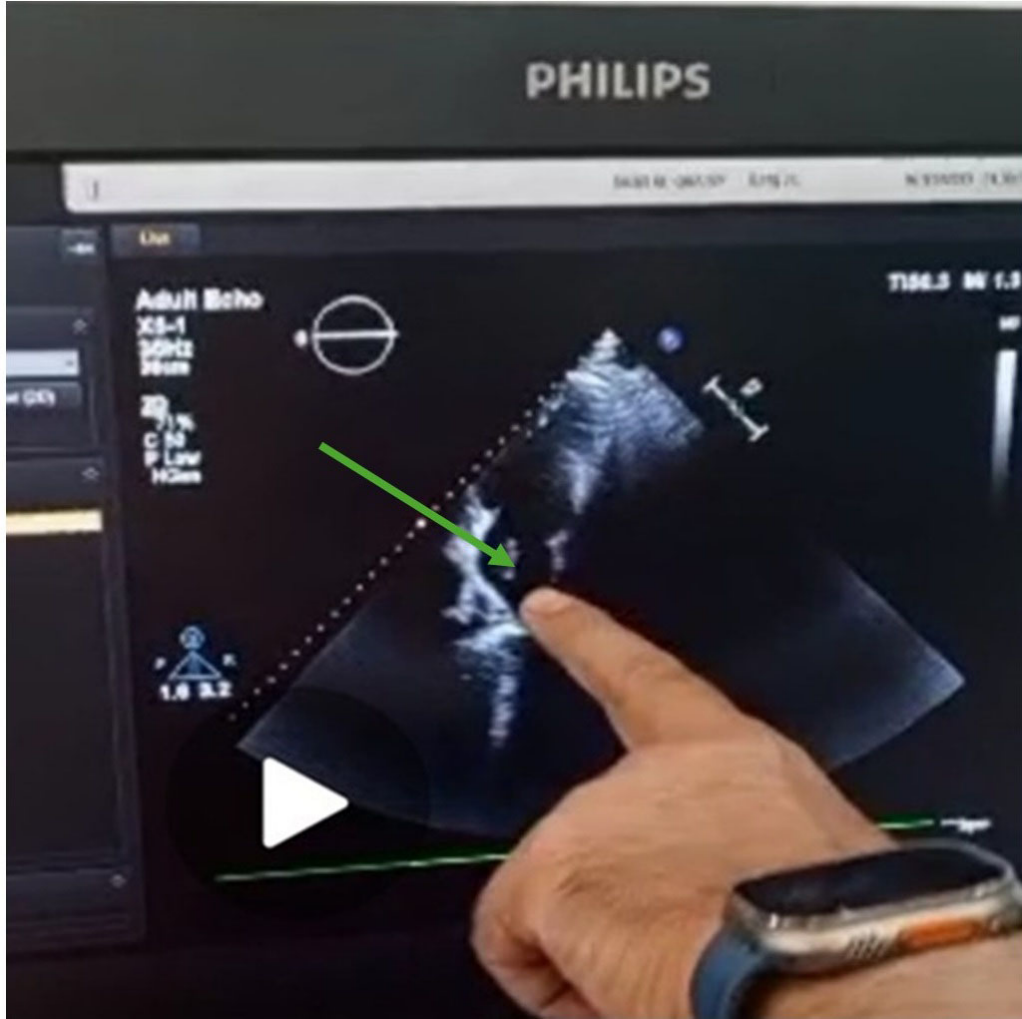
Echocardiography shows left atrial hydatid cyst posterior to the heart containing daughter cysts (colored arrow).

All patients underwent surgery in the Iraqi Center for Heart Diseases/Medical City, Baghdad. Three patients presented with palpitation. One patient presented with ischemic chest pain with elevated cardiac enzyme levels and electrocardiographic (ECG) changes. Another patient presented with weakness and headache due to a brain hydatid cyst, as shown in Table 1. All patients were diagnosed with echo studies with chest CT scans, as shown in Figure 2. Four patients had isolated cardiac hydatid cysts, and the other had brain, cardiac, and spleen hydatid cysts shown in Table 2.

**Table 1.** Clinical presentations.

Palpitation	Ischemic chest pain & elevated S. troponin	CNS manifestation
3	1	1

**Table 2.** Cardiac and extracardiac hydatid.

Isolated	Mixed
4 (cardiac H. C alone)	1 (cardiac, splenic & brain H.C)

Four patients had a left ventricle hydatid cyst, and the other had a left atrial hydatid cyst with showering daughter cyst to the brain, as shown in Table 3.

**Table 3.** Position of H. C in the Heart.

L. Ventricle	L. Atrium
4	1

**Figure 3.**
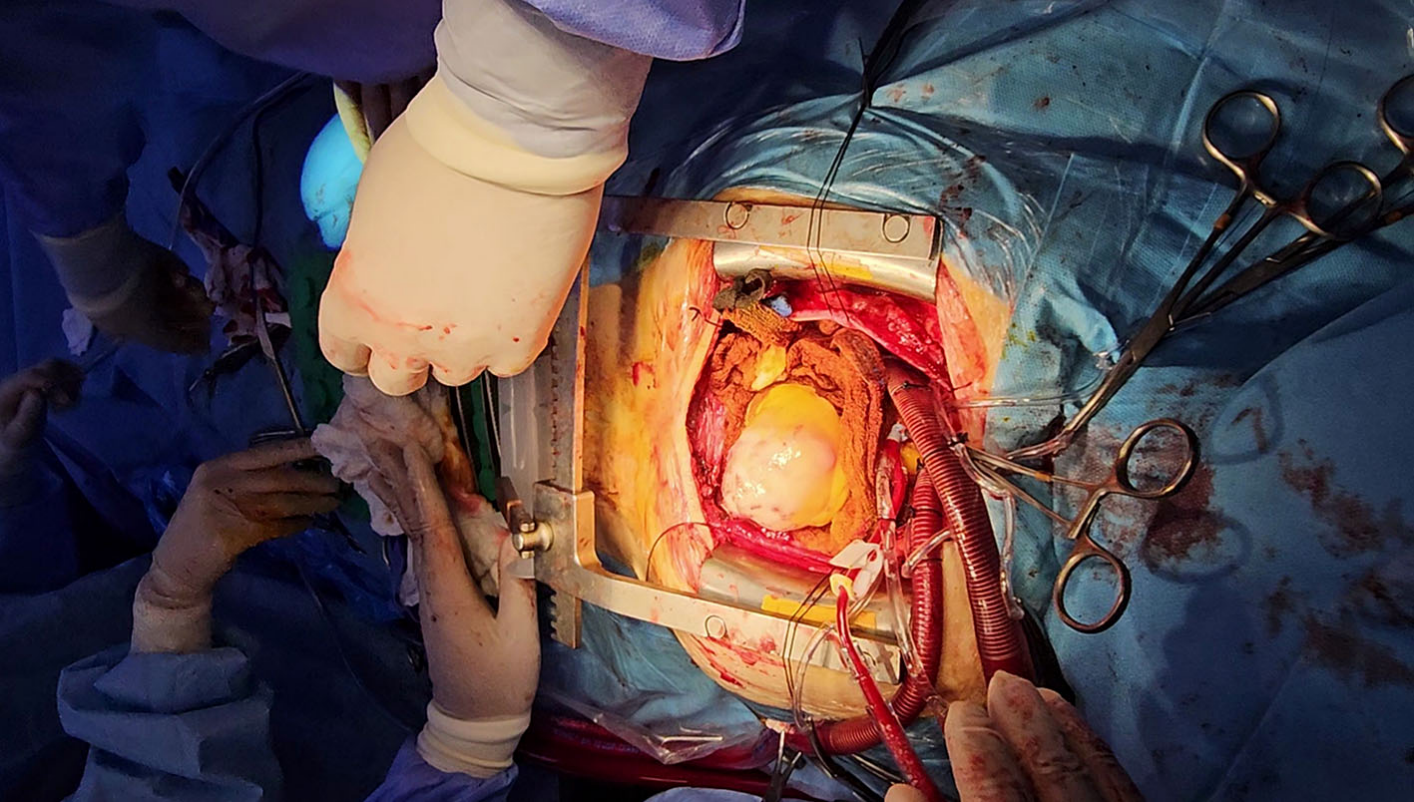
Intact left ventricular hydatid cyst with needle for aspiration of cysts content.

**Figure 4.**
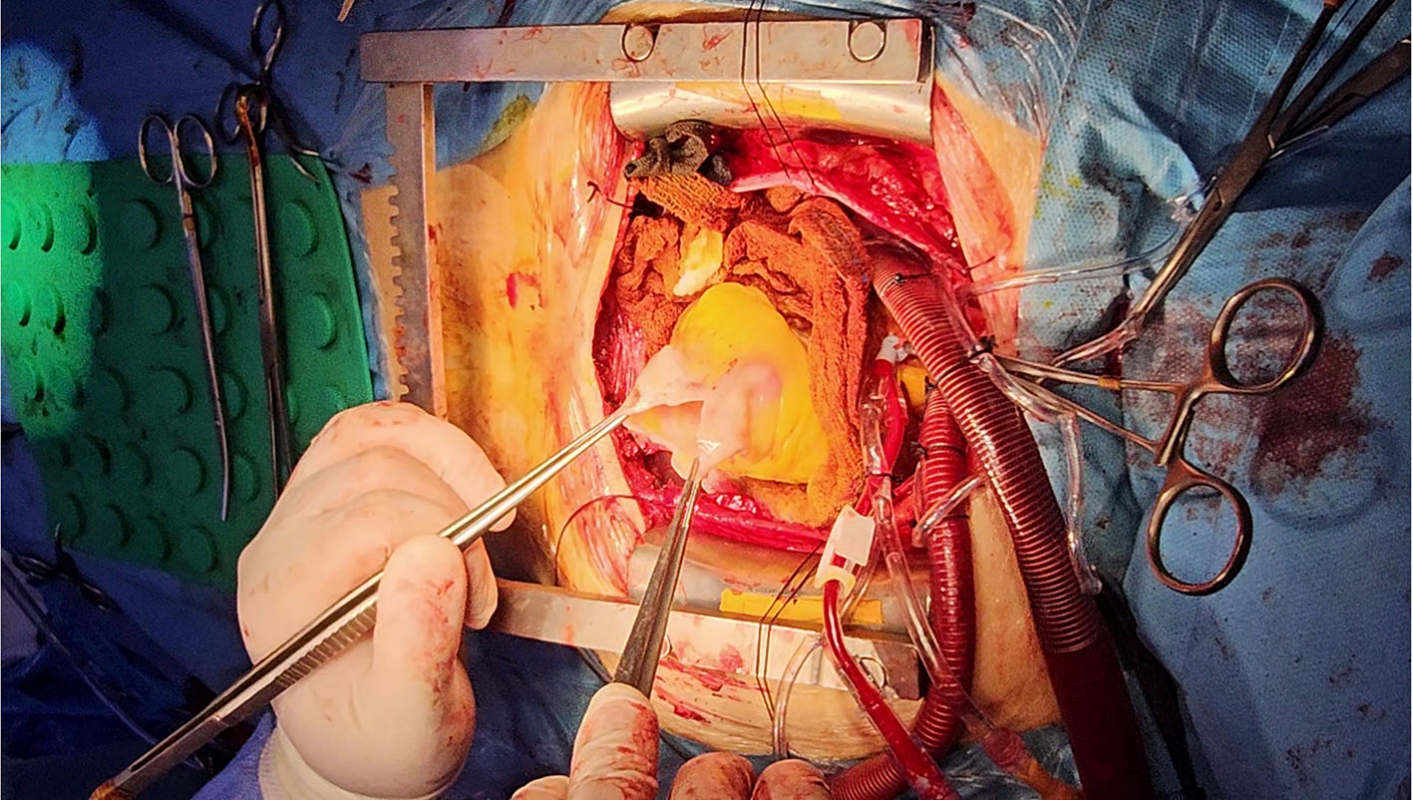
Left ventricular cavity after excision of hydatid cyst.

**Figure 5.**
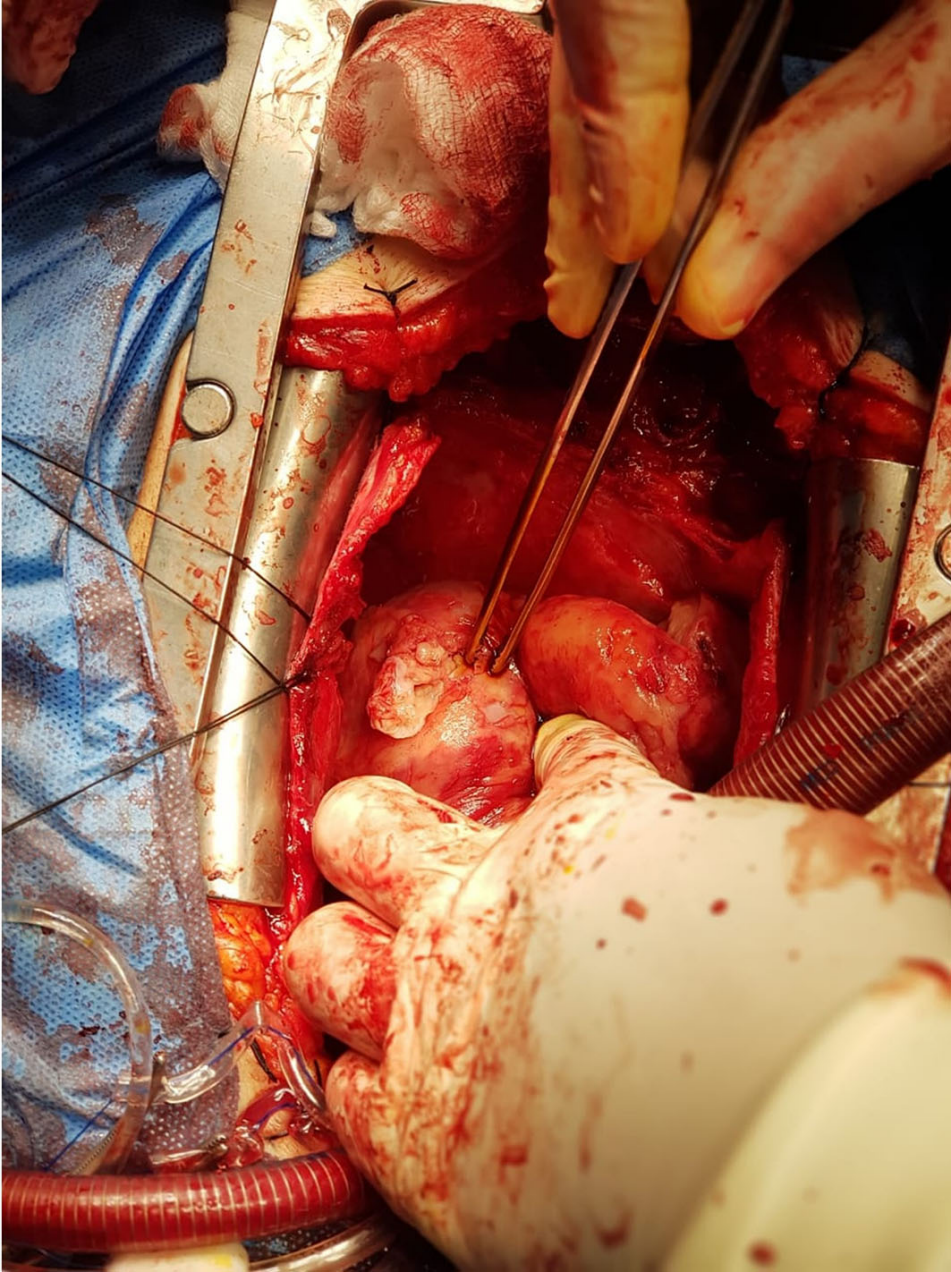
Intrapericardial rapture of cardiac hydatid cyst.

Three patients had ruptured hydatid cysts, two patients had intrapericadial rupture with pericardial adhesion, and one patient had a ruptured cyst in the left atrium (intracardiac ruptured), while the last two patients had intact hydatid cysts, as shown in Figures 3, 4 and 5.

**Figure 6.**
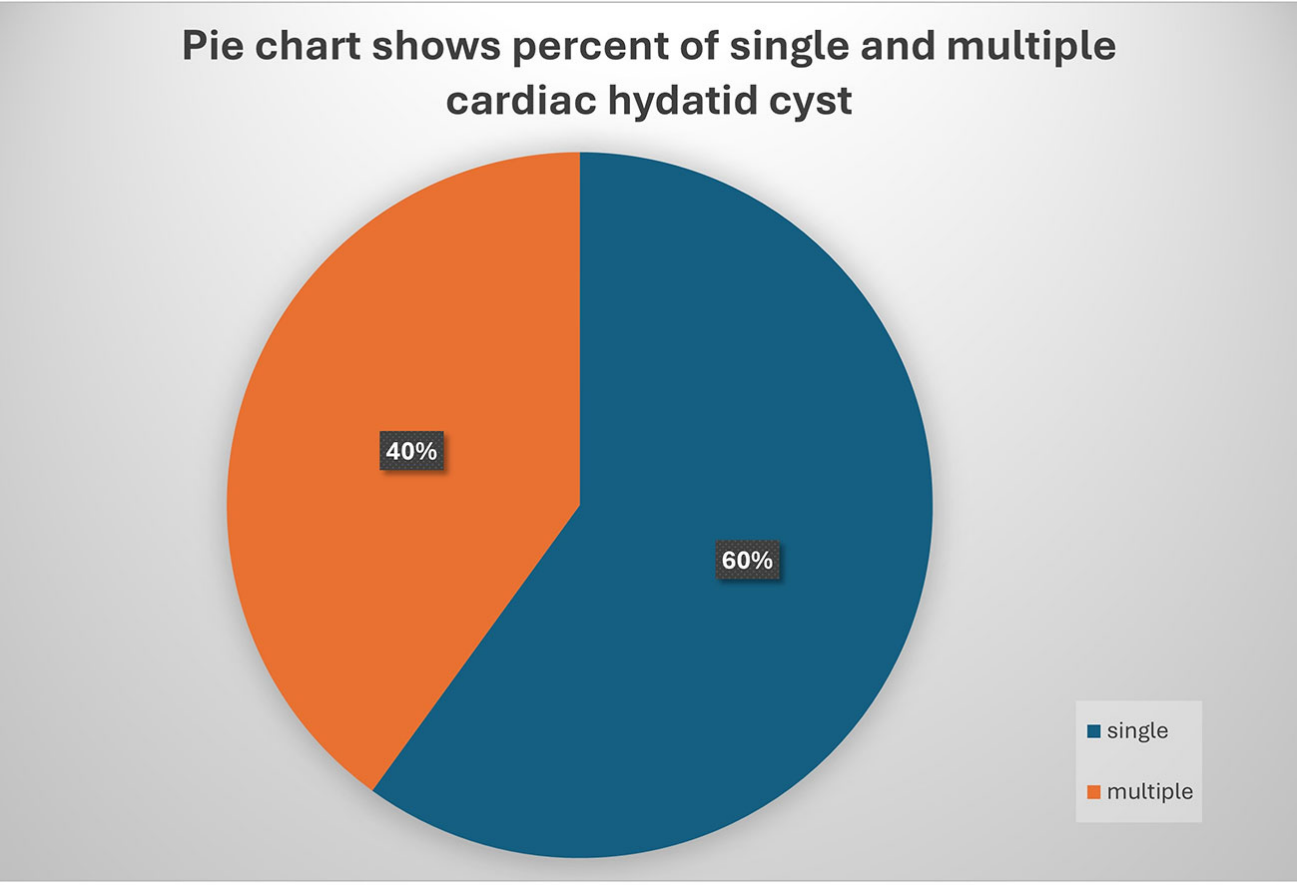
Single and multiple hydatid cyst.

Three patients had single hydatid cysts, whereas the other two patients had multiple cardiac hydatid cysts, as shown in Figure 6.

During the operation, two operations were complicated due to adhesion and extension of the aortic incision (friable tissue due to the inflammatory process), and the other three patients had smooth operations, as shown in Table 4.

**Table 4.** Intraoperative complications.

Complicated operation	Soft operation
2 (adhesion, extension in aortic incision)	3

During the early post Operative period, one patient developed significant bleeding requiring re-exploration on cardiopulmonary bypass and re-suturing of the left ventricular free wall due to elevated. Blood Pressure during recovery and delayed extubation to the 1 ^st^ post operative day, with massive blood transfusion and prolonged ICU stay.

All patients were treated with albendazole (high dose, 400 mg, 1×2). Long-term follow-up in the first two cases for five years showed no recurrence with good cardiac function. Short-term follow-up for the last three cases also showed no recurrence with good cardiac function. Two female patients had a soft pregnancy and labor two years after surgery.

## Discussion

Isolated cardiac hydatid cysts are rare events, ^6^ as contraction of the heart fights vital cysts. Cardiac hydatid cysts appear as makeshift bombs and may rupture at any time; therefore, they should be diagnosed early and treated seriously. There are two types of rupture, intrapericardial rupture and intracardiac rupture, and both complications were present in our cases.

The female: male ratio was 4:1 against Ashur Y. Oraha et al. (2018), ^7^ which was done in Kurdistan (north of Iraq) and included four cases of three male and one female.

In this study we notice the incidence in the last two years 60%, 80% of patients are from urban areas rather than rural areas might be due contaminated food from restaurants, also presence of loose dogs in the cities represents a risk factor to transmission eggs of *E. granuolosis* to cattle, and accidentally human being. Regarding the presentation of patients, 60% presented with palpitation, which agrees with the findings of Ashur Y. Oraha et al. (2018). ^7^ Twenty percent of patients presented with CNS manifestations due to rupture of the left atrial hydatid cyst with a daughter cyst delivered to the brain. While 20% of patients presented with features of IHD due to rupture of a large LV H. C to the pericardium, which stimulates the inflammatory process and pericarditis with myocarditis and elevated cardiac enzymes.

Four patients had LV H. C because coronary circulation is the first station after the blood leaves the heart. This agrees with Oraha et al. (2018). ^7, 8^ Regarding diagnostic investigations of choice, echocardiography and chest CT scan agree with Oraha et al. (2018) ^7^ as the diagnosis of cardiac hydatid cyst is easy with a typical cystic appearance on echocardiography; however, it may rarely be difficult to distinguish it from myxoma. ^9, 10^ Surgery is the treatment modality of choice; all patients underwent median sternotomy open heart surgery, which agrees with the findings of Oraha et al. (2018). ^7^ The patient who had multiple cardiac H. C due to rupture of the main H. C and delivery of daughter cysts to the pericardial space developed extension at the site of aortic cannulation but easily controlled.

During post operative period, patient who developed significant bleeding required re-exploration on cardiopulmonary bypass due to elevated blood pressure, while there was a friable LV wall due to the inflammatory process that caused this separation of the LV wall patch. The mortality rate was zero, with good surgical outcomes.

## Recommendation

• Improve people’s hygiene and sanitation techniques.
• Educate people about hydatid disease and ways of transmission by television programs and posts on social media.
• Slaughterhouse had a responsibility to get rid of the bowel of infected sheep.
• Hysterical disease is the diagnosis of exclusion; some patients had multiple presentations to the ER and were diagnosed with HYS.

## Consent

The patient himself and legal guardian gave written permission to use their clinical data (including photos) in the documentation for the publication.

## Data Availability

All data underlying the result of this article are available as a part of the article and no additional data source is required as this is case series study of rare disease. The completed CARE Checklist supporting this case-series submission has been uploaded to an external public repository (OSF). The file is titled “ **The outcome of cardiac hydatid surgery in the Iraqi center of heart disease”** and is available under a CC0 1.0 Universal license. DOI: **10.17605/OSF.IO/SJUBC** Repository: Open Science Framework (OSF) A full reference to the repository has been included in the Reference list.
