# The Outcome of Cardiac Hydatid Surgery in The Iraqi Center of Heart Diseases : A Retrospective Case-series Study

**DOI:** 10.12688/f1000research.172453.3

**Published:** 2026-06-16

**Authors:** Maath Mohammed Muhsin, Noor Hussain Abady, Aminah Hilal Khallaf

**Affiliations:** 1 Pathology, University of Fallujah, College of medicine, Fallujah, Iraq; 2 Surgery, University of Fallujah, College of medicine, Fallujah, Iraq; 3 Gynecology, Nu'man teaching hospital, Baghdad, Iraq

**Keywords:** Cardiac hydatid cyst, Echinococcosis, Cardiac surgery.

## Abstract

**Background:**

Cardiac hydatid cysts are an uncommon presentation of heart disease and most often affect the left ventricle. The clinical picture may vary considerably and may include arrhythmias and myocarditis, up to a potentially life-threatening embolism of the cystic contents in cases of rupture into a cardiac chamber. Early and proper diagnosis is very important to avoid serious complications such as infection and rupture of the cyst. Although medical treatment may provide a certain degree of control, surgery remains the preferred treatment because it offers good results and a low possibility of recurrence when performed early.

**Methodology:**

This retrospective case-series study examined five patients diagnosed with cardiac hydatid cysts between January 2018 and July 2024. All cases were managed by a qualified cardiac surgeon in Al-Anbar Governorate, Iraq. The effectiveness and safety of surgical management were evaluated on the basis of clinical information, radiographic findings, surgical procedures, and post-operative outcomes.

**Results:**

All five patients successfully underwent excision of the cardiac hydatid cysts. The post-operative course was uneventful in all cases except one patient who developed significant post-operative bleeding that required re-exploration. No deaths occurred, and good clinical recovery was achieved. No incidence of cyst rupture, secondary infection, or cyst recurrence was reported during the follow-up period.

**Conclusions:**

Although uncommon, cardiac hydatid cysts should be included in the differential diagnosis of cardiac masses, particularly in endemic areas. Echocardiography and advanced imaging should be used for early diagnosis to prevent severe complications. Surgery remains the cornerstone of treatment because it offers a high success rate with low risk when performed in a timely and careful manner. The positive results in this series support the importance of timely surgical referral and multidisciplinary management.

## Introduction

Hydatid cysts are parasitic infections mostly caused by *Echinococcus granulosus* and are mostly observed in the liver and lungs. Echinococcosis is a human infection caused by the larval stage of any species of the *E. granulosus* complex, *E. multilocularis*, or *E. vogeli. E. granulosus* complex parasites that cause unilocular cystic lesions are common in areas where livestock are kept in close proximity to dogs. ^1^

*Echinococcus granulosus* has a 2-host life cycle. Dogs and other canines are definitive hosts, whereas sheep and other herbivorous animals are intermediate hosts. Humans serve as an incidental intermediate host (dead end), infected by the ingestion of food contaminated with feces from dogs harboring *E. granulosus* eggs. ^2^

Eggs develop into a larva in the duodenum. The larva pierces the intestinal wall, enters the portal circulation, and is transported to the liver, lungs, or seldom, to other organs. The host immune response attempts to eliminate the parasites after tissue invasion, where an inflammatory reaction occurs around the sites where the parasites are lodged. Although the immune response destroy many parasites, some survive, escape elimination, and continue to develop into hydatid cysts, surrounded by fibrous connective tissue, and become fluid-filled bladder-like cysts, usually in the liver (60-70%, in the right lobe) or lung (20-30%). Hydatid cysts can also occur in other organs such as the spleen and kidney (35%), brain and heart (11.5%), and bones in rare cases. ^2^

The hexacanth embryo (six-hooked, first-stage larva) reaches the heart through the coronary arteries and gives rise to a primary, often solitary, hydatid cyst within the myocardial layers. Secondary cardiac involvement may follow rupture of a primary cyst into the pericardial cavity. In such cases, the resulting cysts are initially superficial and subepicardial but may later extend into the myocardium, where they often become multiple. ^3^

After cardiac hydatid cysts develop, their progressive enlargement may produce characteristic structural and functional effects. Patients from endemic regions who present with an abnormal cardiac shadow on chest radiography (CXR) should, therefore, raise suspicion of cardiac hydatid disease. As the cyst enlarges, it compresses the surrounding myocardium and may displace the coronary vessels, cause rhythm disturbances, and interfere mechanically with atrioventricular valve and ventricular function. Echocardiography remains the preferred imaging modality for the diagnosis of cardiac hydatidosis.

Computed tomography may help confirm the diagnosis and exclude hydatid involvement of the liver, lungs, and brain when uncertainty remains. Case management depends on cyst size, location, symptoms, and the general condition of the patient. Surgery remains the definitive treatment for cardiac echinococcosis. Operative mortality in cystic echinococcosis has generally ranged from 0.5% to 4%, whereas long-term recurrence has ranged from 2% to 25%, with a greater risk after repeat intervention. Older series of cardiac hydatid disease reported operative mortality rates of 4.8% to 10%, although more recent small series have shown better outcomes. Albendazole and mebendazole have demonstrated efficacy against cystic echinococcosis, but albendazole remains the preferred agent due to its superior systemic absorption and greater penetration into hydatid cysts. Medical therapy alone is reserved for inoperable cysts, poor clinical condition, or multiple-organ involvement, and albendazole is also used before and after surgery to reduce recurrence. ^4^ ^,^ ^5^

The study aims to characterize the different presentations of cardiac hydatid cysts in an endemic setting and to emphasize that early diagnosis and timely treatment may prevent major complications, particularly infection and rupture.

## Patients and methods

**Figure 1.**
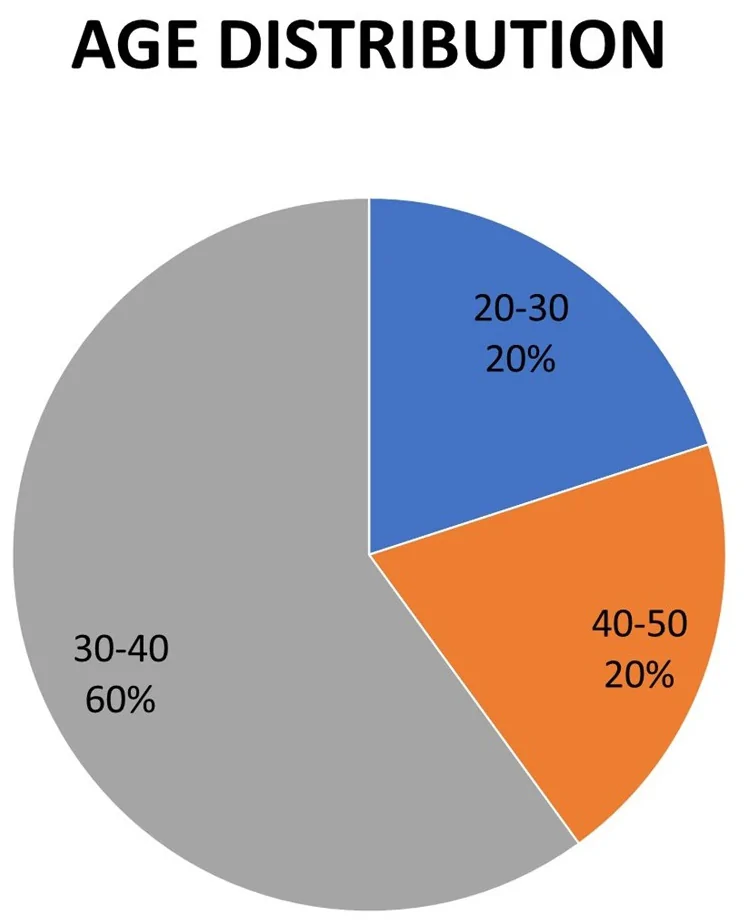
Age distribution of the five patients with cardiac hydatid cysts included in the present case-series.

The present retrospective case-series study included five consecutive patients with cardiac hydatid cysts who underwent surgical treatment at the Iraqi Center for Heart Diseases, Medical City, Baghdad, between January 2018 and July 2024. The series included one case in 2018, one case in 2020, two cases in 2023, and one case in June 2024. Inclusion criteria comprised all patients diagnosed with cardiac hydatid cysts who underwent surgical management at our center during the study period, whereas patients with incomplete records or non-surgical management were excluded. Patient age ranged from 20 to 50 years, and the female-to-male ratio was 4:1, as shown in Figure 1. All patients were referred by a cardiologist to a cardiac surgeon, and all underwent open-heart surgery through median sternotomy with cardiopulmonary bypass and cardioplegic arrest. After exposure of the cyst, the surrounding operative field was isolated with pads soaked in povidone iodine The cyst content was aspirated carefully before excision in order to reduce the risk of spillage. The residual cavity was managed according to cyst location and myocardial involvement. The myocardial defect was repaired according to the size and site of the defect; patch repair was used in cases with a large defect or friable ventricular wall. Synthetic patch was used (PTFE patch).

**Figure 2.**
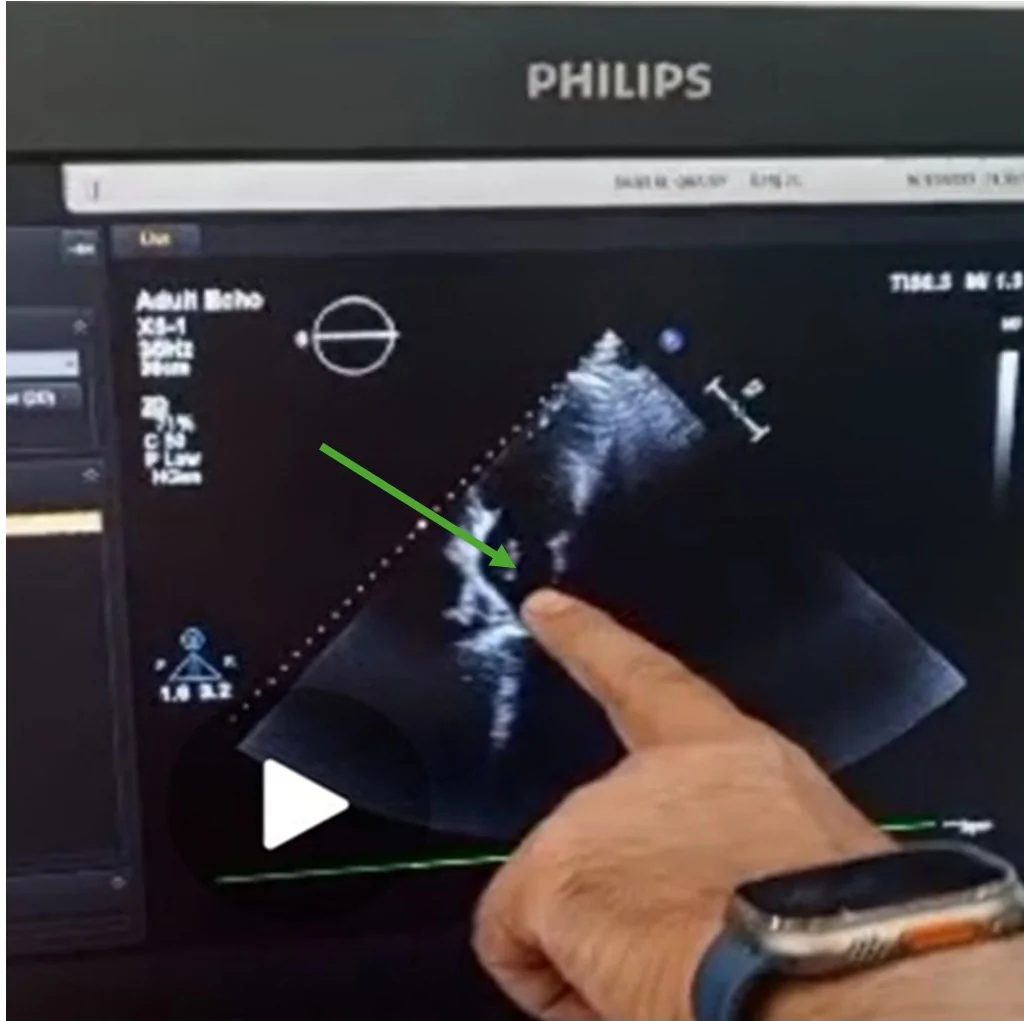
Transthoracic echocardiography showing a left atrial hydatid cyst located posterior to the heart with daughter cysts (colored arrow).

Clinical presentation varied across the series. Palpitation was the most frequent presenting complaint and occurred in three patients. One patient presented with ischemic chest pain associated with elevated cardiac enzyme levels and electrocardiographic changes. One patient presented with weakness and headache secondary to a brain hydatid cyst. Diagnosis was established in all patients by echocardiographic examination and chest computed tomography. Serological testing for echinococcosis was performed in some patients when available. Figure 2 shows an echocardiographic image of a left atrial hydatid cyst located posterior to the heart with daughter cysts. In the left atrial case, the diagnosis favored hydatid cyst rather than myxoma because imaging showed a cystic lesion with daughter cysts and without the typical pedunculated attachment expected for atrial myxoma. Four patients had isolated cardiac hydatid disease, whereas one patient had combined cardiac, splenic, and brain hydatid cysts. In the patient with multi-organ hydatid disease, cardiac surgery was prioritized because of the immediate risk associated with the intracardiac lesion. The extracardiac lesions were managed in a staged manner with postoperative follow-up and adjunct medical therapy.

**Figure 3.**
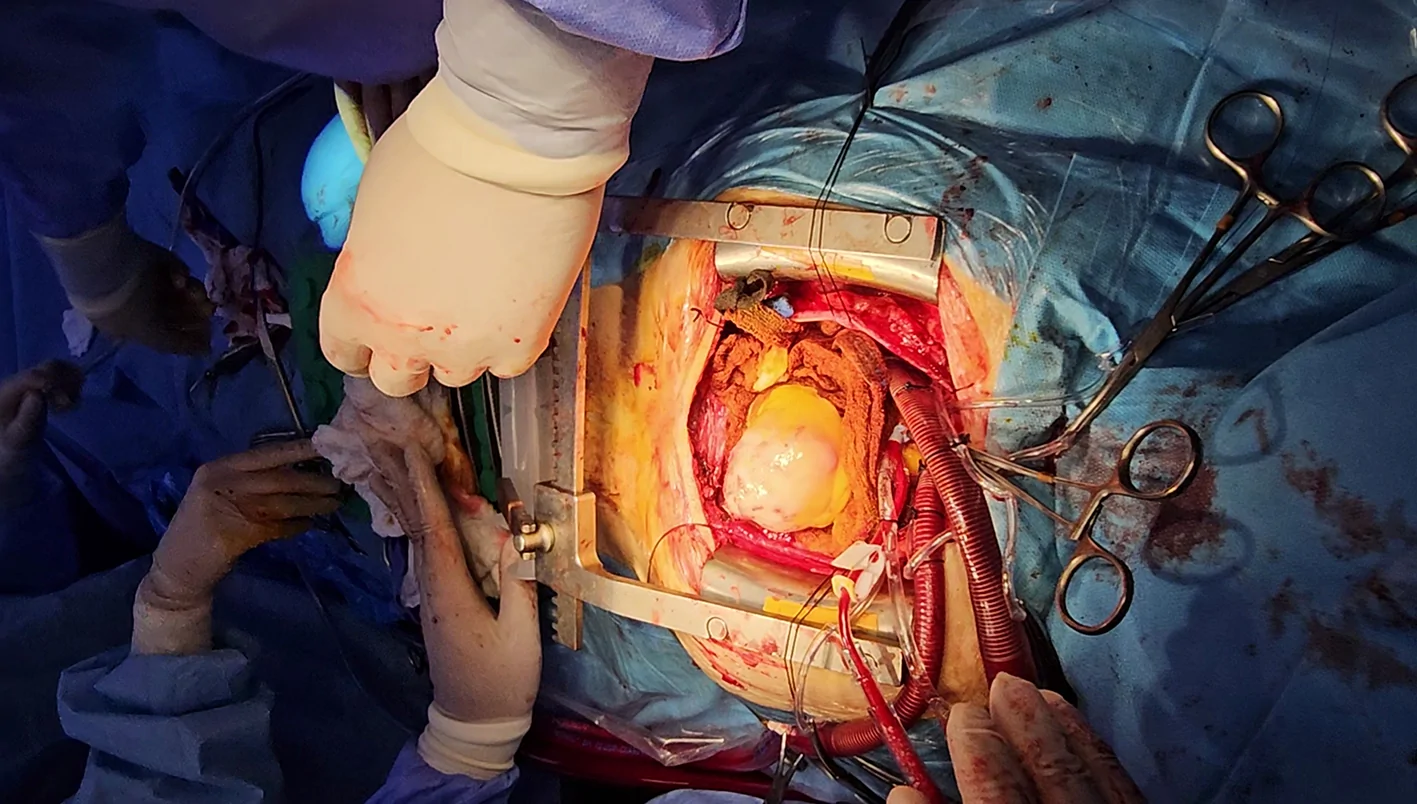
Intraoperative photograph of an intact left ventricular hydatid cyst during careful aspiration of cyst content before excision.

**Figure 4.**
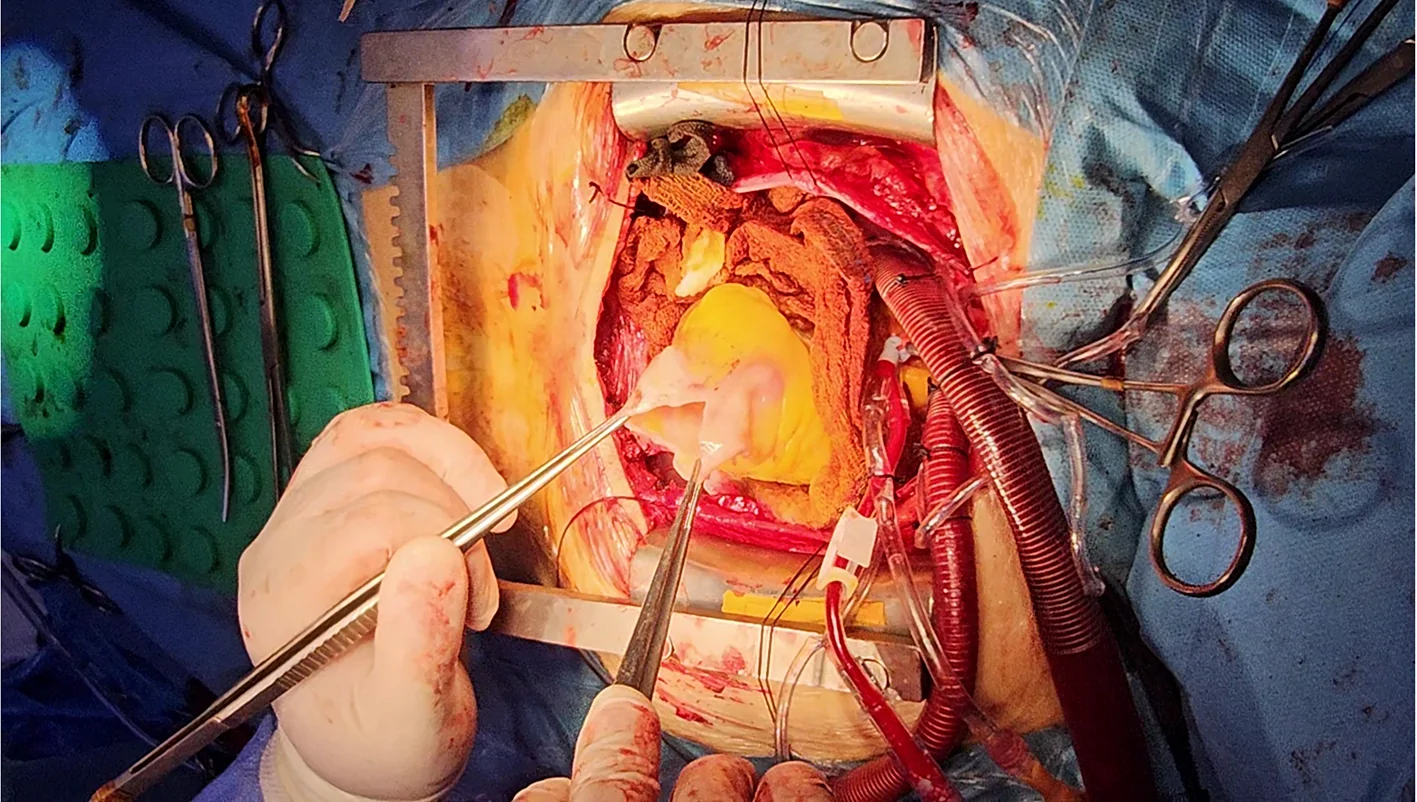
Intraoperative view of the left ventricular cavity after excision of the hydatid cyst.

**Figure 5.**
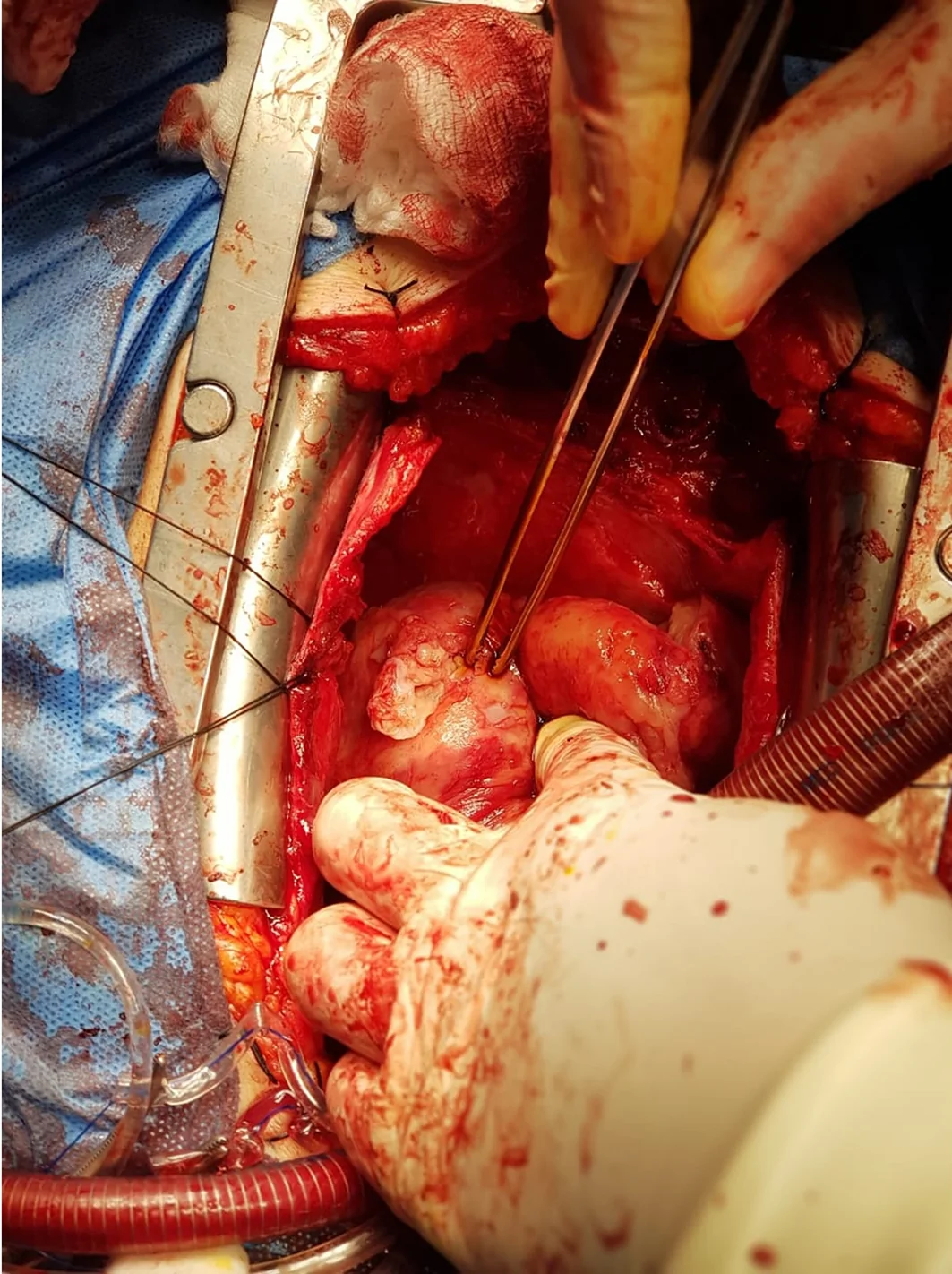
Intraoperative view showing intrapericardial rupture of a cardiac hydatid cyst.

Cardiac involvement affected the left ventricle in four patients. One patient had a left atrial hydatid cyst with daughter cyst dissemination to the brain. Three patients had ruptured hydatid cysts. Two of those patients had intrapericardial rupture associated with pericardial adhesions, whereas one patient had rupture of a left atrial cyst into the cardiac cavity. Two patients had intact cysts. Figure 3 shows an intact left ventricular hydatid cyst during aspiration of the cyst content, Figure 4 shows the left ventricular cavity after cyst excision, and Figure 5 shows intrapericardial rupture of a cardiac hydatid cyst. Three patients had a single cardiac hydatid cyst, whereas two patients had multiple cardiac hydatid cysts.

Operative course was reviewed in all cases, where two operations were complicated by adhesions and extension of the aortic incision, which the operative notes attributed to friable tissue related to the inflammatory process. Three operations proceeded without major intraoperative difficulty. Early postoperative review identified one patient who developed significant bleeding and required re-exploration on cardiopulmonary bypass with re-suturing of the left ventricular free wall. Elevated blood pressure during recovery contributed to delayed extubation until the first postoperative day, massive blood transfusion, and prolonged intensive care unit stay.

Albendazole was administered in all patients at a high dose of 400 mg twice daily. Follow-up of the first two cases extended to five years and showed no recurrence with good cardiac function. Follow-up of the last three cases was shorter, but no recurrence was detected and cardiac function remained good. Two female patients later completed uncomplicated pregnancy and labor two years after surgery. A summary of patient demographics, clinical presentation, cyst characteristics, operative management, follow-up, and outcomes is presented in Table 1.

**Table 1.** Summary of patient demographics, clinical presentation, cyst characteristics, management, follow-up, and outcomes.

Variable	Findings in the present series
Number of patients	5
Study period	January 2018 to July 2024
Year distribution	1 case in 2018, 1 case in 2020, 2 cases in 2023, 1 case in June 2024
Age range	20–50 years
Female:male ratio	4:1
Residence	1 rural, 4 urban
History of sheep contact	None reported
Presentations	Palpitation in 3 patients; ischemic chest pain with elevated cardiac enzymes and ECG changes in 1 patient; weakness and headache due to brain hydatid cyst in 1 patient
Diagnostic tools	Echocardiography and chest CT in all patients
Cardiac involvement	Left ventricle in 4 patients; left atrium in 1 patient
Extracardiac involvement	Isolated cardiac disease in 4 patients; combined cardiac, splenic, and brain hydatid disease in 1 patient
Rupture status	3 ruptured cysts, 2 intact cysts
Type of rupture	2 intrapericardial ruptures with pericardial adhesion; 1 intracardiac rupture in the left atrium
Cyst number	Single cardiac cyst in 3 patients; multiple cardiac cysts in 2 patients
Surgical approach	Open-heart surgery through median sternotomy, cardiopulmonary bypass, and cardioplegic arrest in all patients
Intraoperative course	2 operations complicated by adhesions and extension of the aortic incision; 3 operations without major intraoperative difficulty
Early postoperative event	1 patient developed significant bleeding requiring re-exploration and re-suturing of the left ventricular free wall
Anthelmintic therapy	Albendazole 400 mg twice daily in all patients
Follow-up	First 2 cases followed for 5 years; last 3 cases had shorter follow-up
Outcome	No mortality, no recurrence, and good cardiac function in all cases

## Discussion

Isolated cardiac hydatid cysts are rare events, ^6^ as contraction of the heart fights vital cysts. Cardiac hydatid cysts appear as makeshift bombs and may rupture at any time; therefore, they should be diagnosed early and treated seriously. There are two types of rupture, intrapericardial rupture and intracardiac rupture, and both complications were present in our cases.

The female: male ratio was 4:1, unlike Oraha *et al.* (2018), ^7^ which was conducted in Kurdistan, north of Iraq, and included four cases, three male and one female.

In this study, we noticed that the incidence in the last two years was 60%. Eighty percent of patients were from urban areas rather than rural areas, which might be due to contaminated food from restaurants. The presence of loose dogs in the cities also represents a risk factor for transmission of eggs of *E. granulosus* to cattle and, accidentally, to human beings. Regarding the presentation of patients, 60% presented with palpitation, which agrees with the findings of Oraha *et al.* (2018). ^7^ Twenty percent of patients presented with CNS manifestations due to rupture of the left atrial hydatid cyst with a daughter cyst delivered to the brain. Another 20% of patients presented with features of IHD due to rupture of a large LV H.C to the pericardium, which stimulated the inflammatory process and caused pericarditis with myocarditis and elevated cardiac enzymes.

Four patients had LV H.C because coronary circulation is the first station after the blood leaves the heart. That finding agrees with Oraha *et al.* (2018). ^7^ ^,^ ^8^ Regarding diagnostic investigations of choice, echocardiography and chest CT scan agree with Oraha *et al.* (2018), ^7^ as the diagnosis of cardiac hydatid cyst is easy with a typical cystic appearance on echocardiography. However, it is difficult to distinguish it from myxoma in rare cases. ^9^ ^,^ ^10^ Surgery is the treatment modality of choice; all patients underwent median sternotomy open heart surgery. The patient who had multiple cardiac hydatid cysts after rupture of the main cyst and spillage of daughter cysts into the pericardial space developed extension of the aortic cannulation site during surgery, but the complication was controlled successfully.

During the post-operative period, the patient who developed significant bleeding required re-exploration on cardiopulmonary bypass due to elevated blood pressure, while there was a friable LV wall due to the inflammatory process that caused separation of the LV wall patch. The friability in that case was most likely related to inflammatory involvement of the myocardial tissue by the ruptured hydatid cyst. In similarly fragile cases, reinforcement with a biological patch may offer additional protection against separation at the repair site, although this possibility requires further evaluation. However, the mortality rate was zero, with good surgical outcomes. In our study we use synthetic patch as biological patch was not available in our center at that time. However, the mortality rate was zero, with good surgical outcomes.

The study has several limitations as the sample size was small because cardiac hydatid disease is rare, the study was retrospective, and follow-up duration was not uniform across all patients. Serological testing and advanced imaging were not available in a standardized manner for every case. These limitations reduce the generalizability of the findings, although the series still provides useful clinical and operative observations from an endemic setting.

## Recommendation

• Improve public hygiene and sanitation practices.
• Increase public awareness of hydatid disease and its modes of transmission through television programs, social media, and other health education platforms.
• Slaughterhouses should ensure proper disposal of the viscera and bowel of infected sheep in order to limit further transmission.
• Functional or non-organic diagnoses should be considered only after exclusion of organic causes, especially in patients with repeated presentations to the emergency department in endemic areas.

## Conclusion

Cardiac hydatid cyst is an uncommon but important diagnosis in endemic settings and requires early recognition because delayed treatment may lead to serious complications. The present case-series showed favorable surgical outcomes, with no mortality, no recurrence during follow-up, and good postoperative cardiac function in all patients. Echocardiography and computed tomography played an important role in diagnosis, whereas timely surgical intervention remained central to management. The limited sample size and non-uniform follow-up restrict generalization, but the findings still support early diagnosis and surgical treatment as key components of care in operable cases.

## Consent

The study was based on a retrospective review of hospital-admitted patients who had already received standard clinical care. According to institutional practice, separate ethics committee approval was not required for retrospective analysis of anonymized clinical data. Despite that, ethical approval was obtained from the University of Fallujah.

Written informed consent for treatment had been obtained at hospital admission, and written informed consent for publication of clinical details and images was obtained from the patients or their legal guardians.

## Data Availability

All data underlying the result of this article are available as a part of the article and no additional data source is required as this is case series study of rare disease. The completed CARE Checklist supporting this case-series submission has been uploaded to an external public repository (OSF). The file is titled “ **The outcome of cardiac hydatid surgery in the Iraqi center of heart disease”** and is available under a CC0 1.0 Universal license. DOI: 10.17605/OSF.IO/SJUBC Repository: Open Science Framework (OSF) A full reference to the repository has been included in the Reference list.
