# Supplementary material for: The Outcome of Cardiac Hydatid Surgery in The Iraqi Center of Heart Diseases : A Retrospective Case-series Study
Source: F1000Res. 2026 Jul 11;15:65. Originally published 2026 Jan 16. [Version 4] doi: 10.12688/f1000research.172453.4 (PMC13320123; doi:10.12688/f1000research.172453.4)
Supplement: Peer review history

# Reviewer report: Reviewer response for version 3

**DOI:** 10.5256/f1000research.202338.r494310

**Published:** 2026-06-30

**Authors:** Ali Bilal Ulas

**Affiliations:** 1 Department of Thoracic Surgery, Ataturk University, Erzurum, Turkey

I am pleased with the latest revised version of the article, thank you.

Are enough details provided of any physical examination and diagnostic tests, treatment given and outcomes?

Partly

Is the background of the cases’ history and progression described in sufficient detail?

Partly

Is sufficient discussion included of the importance of the findings and their relevance to future understanding of disease processes, diagnosis or treatment?

No

Is the conclusion balanced and justified on the basis of the findings?

Partly

Reviewer Expertise:

Thoracic Surgery

I confirm that I have read this submission and believe that I have an appropriate level of expertise to confirm that it is of an acceptable scientific standard.

# Response

**Published:** 2026-07-05

**Authors:** د.معاذ محمد

Thank you for your positive feedback. We appreciate your time and thoughtful review of our manuscript. We are pleased that the revised version meets your expectations.

# Reviewer report: Reviewer response for version 3

**DOI:** 10.5256/f1000research.202338.r494309

**Published:** 2026-06-22

**Authors:** Laila Jedidi

**Affiliations:** 1 University of Tunis El Manar, Tunis, Tunisia

-Although improved, the discussion still lacks deeper analysis. for example:

 "Coronary circulation is the first station after the blood leaves the heart" --> this is an oversimplified explanation for left ventricular predominance.

 -Follow-up remains heterogeneous: the first two patients had 5 years of follow-up while the others had much shorter follow-up. therefore, "No recurrence"

 should be interpreted cautiously.

Are enough details provided of any physical examination and diagnostic tests, treatment given and outcomes?

Partly

Is the background of the cases’ history and progression described in sufficient detail?

Yes

Is sufficient discussion included of the importance of the findings and their relevance to future understanding of disease processes, diagnosis or treatment?

Partly

Is the conclusion balanced and justified on the basis of the findings?

Yes

Reviewer Expertise:

General Surgery, Hydatid disease

I confirm that I have read this submission and believe that I have an appropriate level of expertise to confirm that it is of an acceptable scientific standard.

# Response

**Published:** 2026-07-05

**Authors:** د.معاذ محمد

Thank you for your valuable and constructive comments. We appreciate your insightful suggestions.

 We agree that the Discussion section would benefit from a deeper analysis. We will revise the explanation regarding the predominance of left ventricular involvement by replacing the oversimplified statement with a more scientifically accurate and evidence-based discussion supported by relevant literature.

 We also acknowledge your comment regarding the heterogeneous follow-up periods. We will revise the manuscript to clearly state this limitation and modify the statement on recurrence to emphasize that the absence of recurrence was observed only during the available follow-up period and should be interpreted with caution given the variable duration of follow-up among the patients.

 Thank you again for helping us improve the quality and scientific rigor of our manuscript.

# Reviewer report: Reviewer response for version 2

**DOI:** 10.5256/f1000research.198742.r481837

**Published:** 2026-05-06

**Authors:** Ali Bilal Ulas

**Affiliations:** 1 Department of Thoracic Surgery, Ataturk University, Erzurum, Turkey

Dear Authors, thank you for the revisions. As I said before, it’s better to use clinical terms; the phrase makeshift bombs is a bit too dramatic for a scientific paper. Instead of "behaving like makeshift bombs" you may use "posing a high risk of spontaneous rupture and subsequent anaphylaxis". The other one, the expression; contraction of the heart fights vital cysts, is a bit vague. Again istead of it you may try; constant myocardial contractility and high intraventricular pressure create a mechanical environment that limits the growth and attachment of hydatid cysts.

Are enough details provided of any physical examination and diagnostic tests, treatment given and outcomes?

Partly

Is the background of the cases’ history and progression described in sufficient detail?

Partly

Is sufficient discussion included of the importance of the findings and their relevance to future understanding of disease processes, diagnosis or treatment?

No

Is the conclusion balanced and justified on the basis of the findings?

Partly

Reviewer Expertise:

Thoracic Surgery

I confirm that I have read this submission and believe that I have an appropriate level of expertise to confirm that it is of an acceptable scientific standard, however I have significant reservations, as outlined above.

# Response

**Published:** 2026-05-22

**Authors:** د.معاذ محمد

Dear Reviewer,

 Thank you very much for your valuable comments and thoughtful suggestions. We appreciate your recommendation to use more precise clinical terminology and have revised the manuscript accordingly.

1. The phrase “behaving like makeshift bombs” has been replaced with:
  “posing a high risk of spontaneous rupture and subsequent anaphylaxis.”
2. The sentence “contraction of the heart fights vital cysts” has been revised to:
  “constant myocardial contractility and high intraventricular pressure create a mechanical environment that limits the growth and attachment of hydatid cysts.”

These modifications have been incorporated into the revised manuscript to improve scientific clarity and academic tone.

 Thank you again for your constructive feedback, which has significantly improved the quality of our work.

 Sincerely 

 Maath Mohammed

# Reviewer report: Reviewer response for version 1

**DOI:** 10.5256/f1000research.190178.r467674

**Published:** 2026-04-01

**Authors:** Laila Jedidi

**Affiliations:** 1 University of Tunis El Manar, Tunis, Tunisia

This manuscript presents a retrospective case series of five patients undergoing surgical management for cardiac hydatid cysts at a single center in Iraq between 2018 and 2024. The authors describe clinical presentations, imaging findings, surgical approaches, and postoperative outcomes. All patients underwent surgical excision with no mortality and favorable outcomes, although one case required re-exploration for postoperative bleeding. The study highlights the importance of early diagnosis and surgical intervention in this rare condition.

 However, few corrections need to be done

 */Inclusion and exclusion criteria are not clearly define

 */It is unclear whether cases were consecutive

 */No clear description of preoperative protocol, Surgical techniques

 */Follow-up is inconsistent: 5 years for some patients, short term for others.

 */The discussion lacks depth and does not sufficiently place findings in the context of existing literature. There is minimal critical analysis, no discussion of limitations, and no clear clinical implications. ->Expand the discussion with a more comparison with published case series. Discussion of recurrence risk and alternative management strategies.

 */Figures are useful but legends lack details

 */comprehensive patient summary table should be added

 */language issues:  

 -Typographical errors: "operatve" → operative  "reccurrence" → recurrence

 "rapture" → rupture

 -Unclear terms: soft pregnancy, Hysterical disease, cardia hydatid, makeshift bombs

 -"successfully underwent successful excision": repetition

 */Which scolicidal agent was used?

 */Were serological tests performed?

 */How was the multi-organ hydatid case managed?

 */How was the myocardial defect repaired?

Are enough details provided of any physical examination and diagnostic tests, treatment given and outcomes?

Partly

Is the background of the cases’ history and progression described in sufficient detail?

Yes

Is sufficient discussion included of the importance of the findings and their relevance to future understanding of disease processes, diagnosis or treatment?

Partly

Is the conclusion balanced and justified on the basis of the findings?

Yes

Reviewer Expertise:

General surgery; clinical research; management of hydatid disease.

I confirm that I have read this submission and believe that I have an appropriate level of expertise to confirm that it is of an acceptable scientific standard, however I have significant reservations, as outlined above.

# Response

**Published:** 2026-04-11

**Authors:** د.معاذ محمد

Dear Reviewer,

 We sincerely thank you for your valuable, thoughtful, and constructive comments. Your feedback greatly helped us improve the structure, clarity, and scientific quality of the manuscript. We carefully revised the paper in response to all points raised. For ease of evaluation, all modifications in the revised manuscript have been highlighted in yellow.

**Comment 1:** Inclusion and exclusion criteria are not clearly defined.

**Response:**

 Thank you for this important comment. We have now clearly defined the inclusion and exclusion criteria in the Patients and methods section. Inclusion criteria comprised all patients diagnosed with cardiac hydatid cysts who underwent surgical management at our center during the study period, whereas patients with incomplete records or non-surgical management were excluded.

**Comment 2:** It is unclear whether cases were consecutive.

**Response:**

 We appreciate this point. We have now clarified that the series included five consecutive patients treated during the study period.

**Comment 3:** No clear description of preoperative protocol and surgical technique was provided.

**Response:**

 Thank you for this valuable comment. We have expanded the Patients and methods section to include the diagnostic work-up with echocardiography and chest computed tomography, serological testing when available, the use of albendazole, and the operative technique. The revised manuscript now specifies median sternotomy, cardiopulmonary bypass, cardioplegic arrest, isolation of the operative field with pads soaked in povidone iodine, careful aspiration before excision, and management of the myocardial defect according to cyst size and site.

**Comment 4:** Follow-up duration is inconsistent.

**Response:**

 We acknowledge this limitation. We have now clarified the follow-up pattern in the text and summarized it in Table 1. The first two cases had follow-up for five years, whereas the last three cases had shorter follow-up. We also added a limitations paragraph in the Discussion to acknowledge the non-uniform follow-up duration.

**Comment 5:** The Discussion lacks depth and critical analysis.

**Response:**

 We appreciate this comment. The Discussion include comparison with previous case series, interpretation of rupture patterns and operative complications, the role of early diagnosis and surgery, and a dedicated paragraph on study limitations and generalizability.

**Comment 6:** Figure legends lack details.

**Response:**

 Thank you for pointing this out. We revised the figure legends to make them more informative by adding the imaging modality, the relevant anatomical findings, and intraoperative context where appropriate.

**Comment 7:** A patient summary table should be added.

**Response:**

 We agree with this helpful suggestion. We added Table 1, which summarizes patient demographics, clinical presentation, cyst characteristics, operative management, follow-up, and outcomes.

**Comment 8:** Multiple language and typographical issues were identified.

**Response:**

 Thank you for noting this. The manuscript has undergone careful language revision, and spelling, grammar, terminology, and phrasing were corrected throughout the text to improve clarity and readability.

**Comment 9:** Which scolicidal agent was used?

**Response:**

 We have now clarified in the Patients and methods section that povidone iodine was used intraoperatively to isolate the surrounding operative field before cyst aspiration.

**Comment 10:** Were serological tests performed?

**Response:**

 Thank you for this comment. We have clarified that serological testing for echinococcosis was performed in some patients when available.

**Comment 11:** How was the multi-organ hydatid case managed?

**Response:**

 We have now added a clearer description of that case. Cardiac surgery was prioritized because of the immediate risk posed by the intracardiac lesion, whereas the extracardiac lesions were managed in a staged manner with postoperative follow-up and adjunct medical therapy.

**Comment 12:** How was the myocardial defect repaired?

**Response:**

 We appreciate this question. The revised manuscript now states that the myocardial defect was repaired according to the size and site of the defect, and that patch repair was used in cases with a large defect or friable ventricular wall.

**Comment 13:** Background and progression are sufficiently described.

**Response:**

 We sincerely appreciate this positive assessment.

**Comment 14:** Diagnostic and treatment details are partly sufficient.

**Response:**

 Thank you for this observation. We have incorporated additional diagnostic, operative, and follow-up details to improve completeness and clarity.

**Comment 15:** Discussion and clinical relevance are partly sufficient.

**Response:**

 We appreciate this comment. The Discussion was slightly improved to strengthen the clinical relevance of the findings, especially regarding rupture patterns, diagnosis, operative management, and the importance of early intervention in endemic areas.

**Comment 16:** Conclusion is balanced and justified.

**Response:**

 We thank you for this positive and encouraging assessment.

 We greatly appreciate your constructive comments, which helped us improve the manuscript substantially.

**Yours sincerely,**

**Maath**

# Reviewer report: Reviewer response for version 1

**DOI:** 10.5256/f1000research.190178.r467669

**Published:** 2026-03-27

**Authors:** Ali Bilal Ulas

**Affiliations:** 1 Department of Thoracic Surgery, Ataturk University, Erzurum, Turkey

Which scolicidal agents were used to soak the surrounding operative field before cyst aspiration?

 Was the myocardial defect patched?

 Was the friability purely inflammatory? Do you think a different surgical approach or a biological patch might have mitigated the separation of the LV wall in such a friable environment?

 In your specific case involving the left atrium, what were the specific echocardiographic features that eventually led you away from a diagnosis of myxoma before the CT scan? Were serological tests performed?

 One patient had brain, cardiac, and spleen hydatids. Was the cardiac surgery prioritized, or were these addressed in a staged fashion? The timing of multi-organ hydatid surgery is a major point of debate in our field.

 Paragraphs 4–5 of the Introduction repeat pathophysiological concepts. Consolidate them.

 References 4 and 5 are broken links.

 Mention the institutional review board or ethics committee approval.

 "operatve" should be operative.

 "reccurrence" should be recurrence.

 "rapture" should be rupture.

 "Hysterical disease" in the recommendations likely refers to Hydatid disease?

 “cardia hydatid” is an incorrect anatomical terminology.

 “successfully underwent successful excision” is redundant.

 "fights vital cysts" is unclear.

 “makeshift bombs” is a non-scientific language.

 “soft pregnancy” what does it mean?

Are enough details provided of any physical examination and diagnostic tests, treatment given and outcomes?

Partly

Is the background of the cases’ history and progression described in sufficient detail?

Partly

Is sufficient discussion included of the importance of the findings and their relevance to future understanding of disease processes, diagnosis or treatment?

No

Is the conclusion balanced and justified on the basis of the findings?

Partly

Reviewer Expertise:

Thoracic Surgery

I confirm that I have read this submission and believe that I have an appropriate level of expertise to confirm that it is of an acceptable scientific standard, however I have significant reservations, as outlined above.

# Response

**Published:** 2026-03-27

**Authors:** د.معاذ محمد

Dear Reviewer,

 We sincerely thank you for your detailed and insightful comments. We highly appreciate your time and expertise, and we have carefully addressed all the points raised. The manuscript has been substantially revised to improve clarity, scientific accuracy, and completeness.

 Please find our point-by-point responses below:

 -----------------------------

 Comment 1:

 Which scolicidal agents were used to soak the surrounding operative field before cyst aspiration?

 Response:

 Thank you for this important comment. We have now clarified in the Methods section that [ povidone-iodine] was used to soak the operative field prior to cyst aspiration to prevent dissemination.

 -----------------------------

 Comment 2:

 Was the myocardial defect patched?

 Response:

 We appreciate this question. The myocardial defect was patched only in one case that presented with hydatic cyst involves left ventricle free wall (full thickness involvement), the patch used was synthetic patch (PTFE). This has now been clarified in the surgical technique section.

 -----------------------------

 Comment 3:

 Was the friability purely inflammatory? Could a different approach or biological patch help?

 Response:

 Thank you for this insightful comment. The friability was most likely related to inflammatory changes associated with hydatid cyst involvement (especially in infected one). We agree that alternative approaches, including the use of biological patches, may reduce the risk of myocardial separation in such fragile tissue. This discussion has now been added to the Discussion section.

 -----------------------------

 Comment 4:

 Left atrial case – differentiation from myxoma? Were serological tests done?

 Response:

 We appreciate this important point. The echocardiographic findings included [ cystic nature, presence of septations, lack of typical stalk attachment, and atypical position as myxoma mostly attaches to inter atrial septum], which raised suspicion away from myxoma. However, definitive diagnosis was achieved by CT imaging.

 Serological tests were [not performed], and this has now been clarified in the manuscript.

 -----------------------------

 Comment 5:

 Multi-organ hydatid disease – surgical timing?

 Response:

 Thank you for highlighting this important clinical consideration. In our case, [cardiac surgery was prioritized due to higher risk]. We have now expanded the discussion to address the controversy regarding timing of multi-organ hydatid disease management.

 -----------------------------

 Comment 6:

 Introduction repetition

 Response:

 We agree with this comment. Paragraphs 4 and 5 have been revised and consolidated to avoid repetition.

 -----------------------------

 Comment 7:

 Broken references

 Response:

 Thank you for noting this. References 4 and 5 have been corrected and updated.

 -----------------------------

 Comment 8:

 Ethics approval

 Response:

 We appreciate this important point. A statement confirming approval from the institutional ethics committee has now been added and obtained from university of Fallujah.

 -----------------------------

 Comment 9:

 Language and typographical errors

 Response:

 Thank you for highlighting these issues. All typographical and language errors have been corrected, including:

 - operative

 - recurrence

 - rupture

 - hydatid disease (corrected from "hysterical disease")

 - cardiac hydatid (corrected terminology)

 - removal of redundant phrases

 - revision of unclear or non-scientific expressions

 The manuscript has undergone thorough language editing.

 -----------------------------

 Comment 10:

 Clarity of case history

 Response:

 We agree that more detail was needed. Additional clinical details regarding patient history and disease progression have now been included.

 -----------------------------

 Comment 11:

 Details of examination, diagnostics, treatment, outcomes

 Response:

 These sections have been expanded to provide clearer and more comprehensive details.

 -----------------------------

 Comment 12:

 Discussion adequacy

 Response:

 We acknowledge this limitation. The Discussion section has been significantly expanded to better highlight the clinical relevance and implications of our findings.

 -----------------------------

 Comment 13:

 Conclusion

 Response:

 The conclusion has been revised to ensure it is balanced and fully supported by the findings.

 -----------------------------

 We hope that these revisions have adequately addressed all your concerns and improved the quality of the manuscript.

 Thank you again for your valuable feedback.

 Kind regards,
